# Larval mosquito management and risk to aquatic ecosystems: A comparative approach including current tactics and gene-drive *Anopheles* techniques

**DOI:** 10.1007/s11248-022-00315-9

**Published:** 2022-07-07

**Authors:** Robert K. D. Peterson, Marni G. Rolston

**Affiliations:** Department of Land Resources & Environmental Sciences, Montana State University, Bozeman, MT 59717-3120 USA

**Keywords:** Risk assessment, Larvicide, Culicidae, Anopheline, Malaria, Mosquito control

## Abstract

Genetic engineering of mosquitoes represents a promising tactic for reducing human suffering from malaria. Gene-drive techniques being developed that suppress or modify populations of *Anopheles gambiae* have the potential to be used with, or even possibly obviate, microbial and synthetic insecticides. However, these techniques are new and therefore there is attendant concern and uncertainty from regulators, policymakers, and the public about their environmental risks. Therefore, there is a need to assist decision-makers and public health stewards by assessing the risks associated with these newer mosquito management tactics so the risks can be compared as a basis for informed decision making. Previously, the effect of gene-drive mosquitoes on water quality in Africa was identified as a concern by stakeholders. Here, we use a comparative risk assessment approach for the effect of gene-drive mosquitoes on water quality in Africa. We compare the use of existing larvicides and the proposed genetic techniques in aquatic environments. Based on our analysis, we conclude that the tactic of gene-drive *Anopheles* for malaria management is unlikely to result in risks to aquatic environments that exceed current tactics for larval mosquitoes. As such, these new techniques would likely comply with currently recommended safety standards.

## Introduction

For the past 20 years, malaria in much of sub-Saharan Africa has primarily been managed by indoor residual treatments of insecticides, long-lasting insecticidal bednets, and artemisinin-based combination therapy (WHO 2020; Zhou et al. 2020). Although these tactics have been remarkably successful in lowering malaria deaths, these gains are threatened by resistance, persistence, and resurgence. Consequently, the World Health Organization (WHO) has called for the research, development, and use of alternative tactics for malaria management to maintain and improve on the successes in recent years (Derua et al. 2019; WHO 2020; Zhou et al. 2020; Antonio-Nkondjio et al. 2021).

Existing and new technologies for mosquito and malaria management pose benefits and risks to human health and ecosystems. Genetically engineered mosquitoes represent a promising tactic for reducing human suffering from malaria. This technology includes gene-drive approaches that suppress populations of specific mosquito species (often referred to as population suppression strategies), such as *Anopheles gambiae* (sensu lato), the vectors of *Plasmodium* spp., the pathogen that causes malaria. Another approach known as population modification does not reduce mosquito populations, but, rather, it limits the ability of mosquitoes to transmit *Plasmodium* spp. but otherwise does not intentionally affect the mosquitoes (Bier 2021).

Currently, research and development of a gene-drive system for population suppression using the the *doublesex* locus (*dsxF*^*CRISPRh*^) has shown promise in experiments with caged, laboratory populations of *An. gambiae* (Kyrou et al. 2018; Connolly et al. 2021; Hammond et al. 2021). Other forms of gene drive are also being researched, including integral gene drives, daisy-chain gene drives, and toxin-antidote recessive embryo (TARE) drives (Nash et al. 2018; Noble et al. 2019; Champer et al. 2020).

The techniques currently being researched that suppress or modify populations of *An. gambiae* have the potential to be used with or even possibly obviate microbial and synthetic organic insecticides. However, these technologies are new and therefore there is attendant concern from opinion leaders, regulators, policymakers, and the general public about their environmental risks (Scudellari 2019; Teem et al. 2019; Connolly et al. 2021). Consequently, there is a pressing need to assist decision-makers and public health stewards by objectively assessing the risks associated with relevant mosquito management tactics so that the risks can be compared to each other as a basis for informed decision making (United Nations 2020).

The optimal way to accomplish this is by using the science-based framework of risk assessment (NRC 1983, 1996, 2009), specifically comparative risk assessment. The purpose of comparative risk assessment is to qualitatively and quantitatively compare different environmental risks for the purpose of improved decision-making (e.g., Peterson and Arntzen 2004; Peterson and Shama 2005; Peterson 2006; Peterson et al. 2006; Davis et al. 2007; Davis and Peterson 2008; Schleier et al. 2008; Schleier and Peterson 2013; Raybould and Macdonald 2018).

In workshop exercises associated with the use of gene-drive mosquitoes in Africa for malaria management, participants identified general protection goals and possible pathways of harm (Roberts et al. 2017; Teem et al. 2019; Connolly et al. 2021). In particular, the groups identified human and animal health, biodiversity, and water quality as major protection goals. Consequently––as one example––it is imperative to understand and communicate the risks of mosquito management tactics to aquatic environments and water quality, including risks to people and other non-target organisms. Therefore, our scope in this paper is to discuss these risks focusing on stressor identification and effects assessment of using gene-drive mosquitoes for malaria management compared to existing non-gene-drive larviciding tactics (i.e., tactics directed at larval mosquitoes). We define “water quality” broadly as that which includes the abiotic and biotic characteristics that determine its suitability for a particular purpose, including consumption by people and other animals (USNOAA 2021).

## Approach and risk characterization

For the purposes of this paper, we define risk assessment as a formalized basis for the objective evaluation of risk in which assumptions and uncertainties are considered and presented (NRC 1983, 1996, 2009; National Academies of Sciences, Engineering, and Medicine 2016; WHO 2017). Both human-health and ecological risk can be described in quantitative terms as a function of effect (in many cases “toxicity”) and exposure (NRC 1983). Risk assessment, therefore, is arguably the most established, robust, and science-based method available to estimate risk. Consequently, it is a powerful tool for evidence-based societal decision-making.

Risk assessment typically uses a tiered modeling approach extending from deterministic models (tier 1) based on conservative assumptions to probabilistic models (tier 4) using refined assumptions (SETAC 1994). Conservative assumptions in lower-tier assessments represent overestimates of effect and exposure; therefore, the resulting quantitative risk values typically are conservative and err on the side of safety.

Although terminology may vary, risk assessments typically follow these steps: (1) problem formulation, (2) analysis phase, and (3) risk characterization (NRC 1983, 1996, 2009; SETAC 1994; EFSA 2010; National Academies of Sciences, Engineering, and Medicine 2016; EFSA et al. 2020). The problem formulation establishes the goals, breadth, and focus of the assessment, the analysis phase has an effects assessment and an exposure assessment, and the risk characterization is a consideration of the joint property of effect and exposure to determine risk or what additional data are needed to calculate risk or refine risk estimates (USEPA 1998a). The effect assessment often includes an identification of the stressor and dose–response or density-response relationships. A stressor (also referred to as a hazard) is the entity that has the inherent ability to cause harm, whether it be a substance, organism, or activity.

On first glance, the risk assessment framework may not seem well aligned with this particular system and question because gene-drive mosquitoes for malaria management are still in research and development stages. Therefore, there is little to no experiential information on potential stressors, effects, and exposure. However, the stepwise nature of risk assessment allows for a logical process whereby risk issues can be presented, compared, and considered (Peterson and Arntzen 2004; Wolt et al. 2010; Raybould and Macdonald 2018; Raybould et al. 2019; Romeis et al. 2020). In addition, genetically engineered *Aedes aegypti* mosquitoes have been produced, assessed for risks, approved by regulatory agencies, and commercially used (Harris et al. 2011; MCTI-CTNBio 2014; Carvalho et al. 2015; USFDA 2016; USEPA 2020a, b), although the techniques and modes of action are different than what is being developed for gene-drive *An. gambiae*. This demonstrates, however, that risk assessment and regulatory approaches are amenable to genetically engineered mosquitoes. This paper will explore via a comparative, qualitative risk assessment framework the risks of using existing larvicides versus those of gene-drive mosquitoes to aquatic environments.

### Conduct of the assessment

The fact that there currently are no gene-drive systems for malaria management that are sufficiently advanced to be presented to regulatory authorities presents fundamental constraints on the thoroughness of risk assessments that can be done. For example, the inherent ability of a genetically engineered protein to cause harm is not yet known for a gene-drive *An. gambiae*. However, the framework is still valuable because we can focus on the problem formulation and effect assessment (especially stressor identification) (Connolly et al. 2021) and engage in initial comparisons to currently used larvicidal tactics.

By focusing on problem formulation and effects, we can identify potential primary and secondary effects, which are important concepts in ecological risk assessment. We define a primary effect as the stressor acting directly on a receptor. The USEPA (1998a) also terms this a “direct effect”. A secondary effect is when the direct response on a receptor becomes a stressor to another receptor (usually another life stage, species, or abiotic entity). The USEPA (1998a) also terms this an “indirect effect.”

**Fig. 1:**
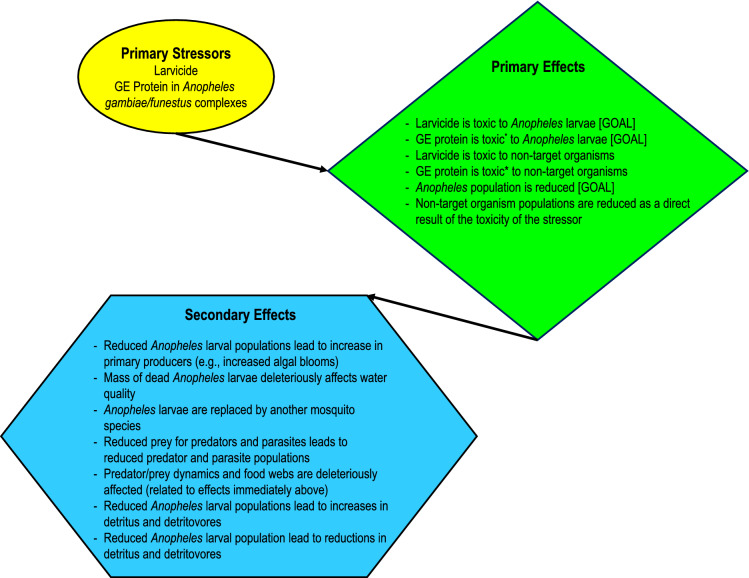
A conceptual map of stressors, primary effects, and secondary effects associated with larvicides and genetically engineered mosquitoes for malaria management in sub-Saharan Africa. *denotes the hypothetical case that the genetically engineered protein is toxic to both the target larvae and non-target organisms even though all current projects suggest that the protein will not be toxic

Previous scoping and problem formulation work on gene-drive mosquitoes has identified potential primary and secondary effects (Roberts et al. 2017; Teem et al. 2019; Connolly et al. 2021) (Fig. 1). Obviously, there will always be limited knowledge of secondary effects posed by a stressor because the possibilities could represent a virtually uncountable number. However, scientifically reasonable and probable secondary effects are a much lower and practically manageable number. Regardless, the concept of primary and secondary effects is important for our purposes because we are dealing with stressors that can be shown to have no or very low inherent toxicity to non-target organisms, including humans. This is especially germane to gene-drive mosquitoes because not only will the engineered proteins most likely be inherently non-toxic to non-target organisms, but they will also most likely be produced by the mosquitoes and will be very low concentrations in the environment.

### Comparative risk assessment

An obvious advantage of comparative risk assessment is that we can evaluate if the new tactic (in this case, gene-drive mosquitoes) has the potential to pose increased risk compared to current tactics (in this case, larvicides). Although obvious, this ability is underused, but is particularly powerful because it allows risk to be evaluated within the context of existing management systems for pests. Comparative risk assessment is also fundamental as a starting point in the safety assessment of genetically engineered organisms, termed “substantial equivalence” (Codex Alimentarius Commission 2003). Furthermore, this concept is embedded in the safety standard suggested by James et al. (2020), which recommends that gene-drive mosquitoes should be released in the field only if they “…will do no more harm to human health than wild-type mosquitoes of the same genetic background and no more harm to the ecosystem than other conventional vector control interventions.”

### Larvicides as the comparator

Because larvicides are the comparator in this assessment, some background on this mosquito management tool is warranted. When used according to product labels, current larvicides will deleteriously affect some aquatic non-target organisms (discussed in detail below). However, these effects most likely will not produce unacceptable risks according to current regulatory thresholds (USEPA 1991, 1998b, 2006). This is because of the regulatory distinction between effects on individuals and populations. In most cases, there will be no effects on populations even though there might be effects on individuals, but there is some evidence of secondary effects on non-target populations with repeated use (Hershey et al. 1998; Lawler 2017; Brühl et al. 2020).

**Fig. 2:**
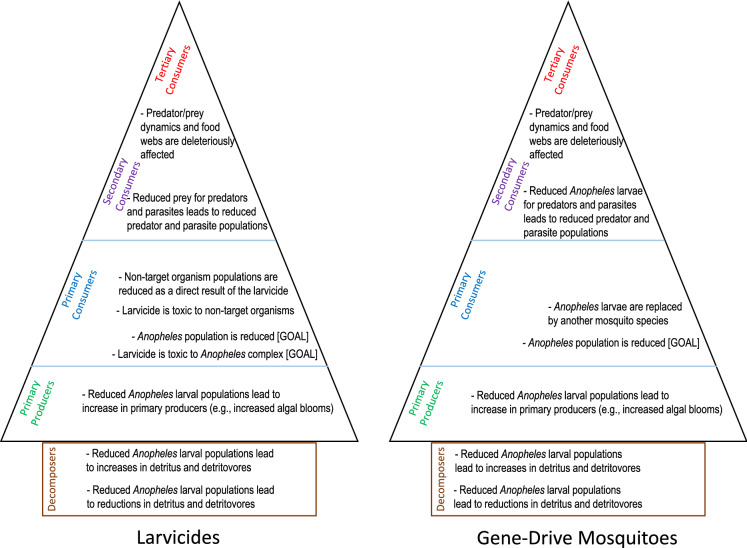
Potential primary and secondary effects of larvicides (left) and gene-drive mosquitoes (right) associated with trophic levels for aquatic ecosystems. Secondary and tertiary consumers are grouped together because the effects would apply to both levels. The “Gene-Drive Mosquitoes” graphic assumes that the active protein is not toxic to non-target organisms. The effects would apply mostly to *Anopheles coluzzii* and *An. funestus* because they are the only species that occupy semi-permanent and permanent water bodies

As mentioned above, the effects assessment in the analysis phase of a risk assessment identifies if a stressor has the inherent ability to cause harm. For conventional larvicides, this is a relatively straightforward process because the stressor is a known toxin and the toxic mode of action is well understood and studied as well as the doses necessary to causes morbidity and mortality (Fig. 2). However, for gene-drive mosquitoes, the transgene may encode proteins that cannot be identified as causing “harm” to any other organism except for the intended effect on the target organism. This fact challenges the notion that complete risk assessments are needed, or can even be done, for some of these products. This is because if there is no inherent ability of the protein to cause harm (i.e., stressor) to any other organism, there is conceptually no need (country-specific regulatory requirements notwithstanding) to engage in the stepwise risk assessment process in which estimates of exposure to the stressor are compared to dose–response relationships (Peterson and Arntzen 2004). Risk assessment traditionally relies on estimating or using actual environmental exposures to the stressor and comparing those to effects to arrive at a characterization of risk––as has been the case for larvicides. For these new tactics such as gene-drive mosquitoes, the problem formulation, stressor identification, and effect assessment arguably will be more important (Fig. 2) to the final estimate of risk.

The purpose of our paper is to comparatively examine issues associated with the risks to water quality from current vector management tactics and from gene-drive mosquito tactics. Although gene-drive mosquito systems for malaria management are still in research and development stages with several engineered genes being investigated, it is highly likely that the resulting proteins will not have conventional insecticidal properties. As such, they should not impose risks on water quality and non-target aquatic organisms that are greater than current larvicides. Indeed, the risks might be appreciably lower (Fig. 2). However, it is important to stress that in most cases gene-drive mosquitoes will be used within an existing Integrated Pest Management (IPM) system for malarial mosquito management (WHO 2017, 2020). Therefore, multiple tactics such as larvicides and gene-drive mosquitoes will be used concurrently and assume that implementation within an IPM system has as a goal ensuring that risks from all tactics are acceptable.

A notable difference between current larvicides and gene-drive mosquitoes is that the mosquitoes (stressor) can multiply in the environment (up to the point at which total population numbers decline over time, which is the purpose of the population suppression gene-drive tactic). This attribute should be part of the risk assessment, but its uniqueness should not be construed as necessitating a separate risk assessment. Indeed, and arguably, risk assessments primarily should be based on the effects of and exposure to the product, not the process by which the product was produced.

### Choice of larvicides

Unlike gene-drive mosquitoes, there is a relatively large amount of data on toxicity and exposure for conventional larvicides. This is because of global regulatory requirements for chemical and biological pesticides as well as years of commercial use after the pesticides have been registered (WHO 2013, 2017). In this paper, we discuss the larvicides methoprene, *Bacillus thuringiensis israelensis*, and *Lysinibacillus sphaericus* (= *Bacillus sphaericus*) to provide examples of risk issues associated with current products. Although there are other larvicides, such as monomolecular films, pyriproxyfen, spinosad, diflubenzuron, temephos, and novaluron, we will not evaluate these out of concerns for brevity, because they are either not currently used for mosquito management in Africa, or because they are unlikely to be used in the near future (Choi et al. 2019; Derua et al. 2019). Similarly, we will not evaluate biological controls, such as larvivorous fish.

### Target species: the *Anopheles gambiae* and *Anopheles funestus* species complexes

Knowledge of habitat and food preferences of the two main species complexes of malaria mosquitoes in Africa is essential because the ecology of these species is critical to understand when assessing primary and especially potential secondary effects. Although we focus on the *An. gambiae* complex for most of this paper, because of the current status of gene-drive research and development, we also are including the *An. funestus* complex because of its importance in malaria transmission and possible future targeting efforts.

Four primary malaria vectors belong to two main mosquito complexes in Africa. The *An. gambiae* complex is comprised of nine species (Sinka et al. 2012; Barrón et al. 2019) and the *An. funestus* complex has 13 species (Ogola et al. 2018). Three of the most important vectors occur within the *An. gambiae* complex (or *An. gambiae* sensu lato (s.l.)): *An. gambiae* sensu stricto (s.s. or S-form), *An. arabiensis*, and *An. coluzzii* (M-form).

Larvae of *An. gambiae* and *An. arabiensis* exploit similar habitats. Both species prefer small, sunlit, temporary, vegetation-free habitats, which are common during the rainy season (Githeko et al. 1996; Gimnig et al. 2001; Koenraadt et al. 2004). Although both anopheline species develop quickly in warm water, a strategy which prevents desiccation in their ephemeral habitats, *An. arabiensis* is better adapted to hot, dry conditions (Githeko et al. 1996), developing approximately one week faster than *An. gambiae* (Schneider et al. 2000). However, the eggs and first instars of both species are relatively resistant to desiccation (Beier et al. 1990; Koenraadt et al. 2003).

Larvae of these two species adapt quickly to temporary, anthropic habitats. During the rainy season, human-made breeding sites include temporary pools created during construction (Khaemba et al. 1994), borrow pits, drinking water vessels, and tire ruts (Gimonneau et al. 2012; Etang et al. 2016). During the dry season, preferred anthropic habitats include brick-making pits (Carlson et al. 2004) and permanent dams (Khaemba et al. 1994). Other production sites consist of early-season rice fields without well-developed vegetation and wells.

*Anopheles coluzzii* and *An. funestus* are also primary malaria vectors in Africa, and they exploit very different habitat types than *An. gambiae* and *An. arabiensis*. Larvae of these species are associated with large, permanent, complex, and stable habitats (Etang et al. 2016). They are commonly found in water bodies dominated by floating plants, overhanging vegetation, and algae and are tolerant of shade (Gimnig et al. 2001; Gimonneau et al. 2012). Preferred habitat includes slow-moving water along rivers and natural ponds (Gimnig et al. 2001), as well as water bodies related to anthropogenic activities such as mature rice fields. The rate of development for *An. coluzzii* is slower, but this species exhibits strong predator-avoidance behavior, an important strategy because predators are more common in the permanent, complex habitats where they occur (Gimonneau et al. 2010).

Abundance of *An. coluzzii* and *An. funestus* peaks during and immediately after the rainy season (Gimonneau et al. 2012), and Kudom (2015) documented that *An. coluzzii* larvae can coexist with *An. gambiae* in temporary habitats such as footprints and tire tracks during this period. However, they are sustained throughout the dry season by breeding in permanent water bodies with high levels of organic material (Kudom 2015). In fact, populations of many anophelines increase early in the dry season, when larval habitats are more stable and less prone to flooding (Kweka et al. 2012, 2015). Warm, ephemeral pools tend to have greater exposure to sunlight, which supports the growth of microorganisms and provides an important food resource for foraging larvae (Minakawa et al. 1999; WHO 2013; Kweka et al. 2015).

### Larvicides: methoprene

Methoprene is a chemical that mimics the juvenile hormone of certain insects. It hinders normal maturation of early mosquito instars, and, therefore, larvae that consume methoprene are unable to reach adulthood (USEPA 1991, 2006). Application timing of methoprene is critical; it works best when the insects are at earlier developmental stages (Gordon and Burford 1984) because late instars, pupae, and adults are not affected.

Methoprene degrades quickly in soil, groundwater, exposed water, and vegetation. Half-lives in water range from 30 h in clean water to 60 to 70 h in sewage. As much as 80% will degrade within 13 days after application (USEPA 1991).

The ecotoxicology of methoprene is reviewed thoroughly by Lawler (2017), and therefore we will only summarize here. Methoprene is practically non-toxic to terrestrial vertebrates and amphibians (USEPA 1991; Lawler 2017). However, fish are susceptible to methoprene exposure at relatively high concentrations that exceed application rates for mosquito management (Brown et al. 1998, 2002; Smith et al. 2003; Hurst et al. 2007); it is moderately toxic to rainbow trout, *Oncorhynchus mykiss*, and bluegill sunfish, *Lepomis macrochirus*.

Methoprene is classified as highly toxic to the planktonic crustacean *Daphnia magna*. It has adverse effects on freshwater amphipods, *Gammarus* sp. (Breaud et al. 1977), lobster (Walker et al. 2005), blue crab, *Callinectes sapidus* (Horst and Walker 1999), fiddler crab (Stueckle et al. 2008), shrimp (Brown et al. 1998; Wirth et al. 2001; Ghekiere et al. 2007), a mayfly species, *Callibaetis pacificus*, non-biting midges (Chironomidae), and a dytiscid beetle, *Laccophilus* sp. (Norland and Mulla 1975).

In a long-term study on experimental ponds where each site was treated at three-week intervals six times over a season, Hershey et al. (1998) concluded that methoprene had a negative effect on aquatic insect predators at treated sites. These impacts were considered to be both direct and indirect through food and interaction webs, as the chemical acted to cause mortality to the predator populations, but also decreased the availability of prey. Pinkney et al. (2000) observed that methoprene applied to experimental ponds had no significant impact on non-target arthropods compared to control treatments.

In a reasonable worst-case (i.e., tier-1) risk assessment, Davis (2007) found that acute and chronic exposures to methoprene did not exceed USEPA regulatory levels of concern for *Daphnia magna*, bluegill sunfish, or rainbow trout. In a review focused on environmental safety, Lawler (2017) concluded that the rates of methoprene used for mosquito management have no detectable effects on the majority of freshwater and marine invertebrates evaluated. Further, Lawler (2017) stressed the important distinction between outcomes from laboratory toxicological studies (i.e., effects) and field studies and actual environmental exposures (i.e., risk).

### Larvicides: *Bacillus thuringiensis israelensis*

*Bacillus thuringiensis* (Bt) is a soil bacterium. Its insecticidal property is the result of a crystalline by-product (endotoxin) of sporulation that affects an insect’s microvillar lining when consumed (Mittal 2003). The insecticide most likely creates an infection court for secondary infection by other bacteria that are common in the insect’s midgut (Broderick et al. 2006) as well as other toxic mechanisms (Caccia et al. 2016). Bt is a highly regarded insecticide because its many strains target specific insect species or narrow groups of insects. Consequently, it is well known that Bt endotoxins are practically non-toxic to mammals, fish, and birds (Mittal 2003) and they break down quickly in the environment (USEPA 1998b).

*Bacillus thuringiensis israelensis* (Bti) is the strain of Bt that is used for mosquito management. Bti is practically non-toxic to mammals, birds, and fish (Mittal 2003) and is not persistent (Hajaij et al. 2005), although it is toxic to some aquatic receptors, including non-biting midges (Chironomidae). Ali (1981) found that applications of Bti to experimental ponds significantly lowered numbers of non-target chironomids. At the highest treatment rate of 4,000 g/ha, there was a 54 to 92% reduction in chironomid abundance. In golf-course ponds at a treatment of 3,000 g/ha, there was a 30 to 67% chironomid reduction, but numbers returned to pre-treatment levels 14 days after treatment (Ali 1981). Charbonneau et al. (1994) found that although Bti caused high mortality of chironomids in a laboratory, a much lower and statistically non-significant mortality was observed in the field. Similarly, Duchet et al. (2015) did not observe any effects on two chironomid species and Lagadic et al. (2016) observed no immediate or long-term effects on chironomid community structure after application of Bti.

However, a series of recent studies in Europe suggest repeated use of Bti has secondary deleterious effects on predators (Jakob and Poulin 2016; Poulin and Lefebvre 2018), primarily through reducing chironomid populations. Allgeier et al. (2019) and Brühl et al. (2020) observed significant reductions in adult chironomid emergence rates after Bti applications in mesocosm and field studies. In a microcosm experiment, Bordalo et al. (2021) also observed deleterious effects on stream benthic invertebrates, including chironomids. It is important to note that in many of these studies, the location evaluated received 30 to 50 aerial Bti applications per year, an exceptionally high frequency of application for Bti. However, WHO (2013) has recommendations that include a maximum of 24 applications per year.

Two formulations of Bti had no effect on non-target invertebrates, including the amphipod *Hyalella azteca*, in test ponds that had a Bti concentration of 100 mg/L (Gharib and Hilsenhoff 1988). Milam et al. (2000) found that treatments of Bti were much more damaging to *An. quadrimaculatus* than sentinel species, including *Ceriodaphnia dubia*, *Daphnia magna*, *Daphnia pulex*, and *Pimephales promelas.* In a laboratory assay, Olmo et al. (2016) observed dose–response toxicity in two copepod and three cladoceran species. Hershey et al. (1998) conducted a large-scale study using 27 experimental ponds in Minnesota, USA. The focus of their study was to determine the impact of multiple aerially applied direct applications of granular methoprene and Bti on non-target invertebrates. Bti and methoprene significantly lowered numbers of chironomids, tipulids, ceratopogonids, and brachycerans in treatment ponds. Disruption of food webs and interaction webs was hypothesized to have occurred in many of these reductions because predators seemed to decline with prey. However, populations rebounded in the years after the treatments. Niemi et al. (1999) found changes in insect diversity in Bti-treated ponds, and reduced total insect numbers in ponds treated with both methoprene and Bti. Lawler et al. (1999) found that Bti and methoprene had no measurable impact on sentinel amphipods in ephemeral mangrove swamps on Sanibel Island, Florida, USA when treated with Bti granules at 5.6 kg/ha and a methoprene liquid formulation applied at 10.65 ml AI/ha for the control of *Aedes taeniorhynchus*. Davis and Peterson (2008) did not observe any overall deleterious effects on non-target arthropods in a field experiment with a single application of Bti.

Ecological effects have been noted for Bti used for black fly and mosquito management. Merritt et al. (1989) observed few changes in indices used to measure treatment effects of Bti used for black fly management in a Michigan river. Drift samples taken at a control and treatment site did not differ for chironomids, baetids, gammarids, or hydropsychids, but there were some treatment effects on perlid stoneflies and elmid beetles. Similar results were observed in 10 stream trials measuring stream insect density of selected taxa (Lawler 2017). Molloy (1992) observed that Bti applied for black fly control within a New York stream affected filter-feeding chironomids, but not surface-dwelling or tube-dwelling members of the same family. Caddisflies and mayflies showed no positive or negative response to Bti treatments.

### Larvicides: *Lysinibacillus sphaericus*

*Lysinibacillus sphaericus* (= *Bacillus sphaericus*) is a soil bacterium that has a similar insecticidal action as Bti (Mittal 2003). For *L. sphaericus*, the insecticidal agent is in the spore cell wall and is a by-product of spore production (Mittal 2003). When the agent is consumed by the mosquito larva, it degrades the lining of the midgut. The insecticide is more effective against *Anopheles* and *Culex* species than *Aedes* species (Mittal 2003), and it remains more active in eutrophic waters than Bti (Lawler 2017).

Brown et al. (2004) found no toxicity to non-target Australian fauna including the fish *Pseudomugil signifier* and the shrimp *Leander tenuicornis*. Merritt et al. (2005) observed similar results in a three-year study in two habitats in which 138 invertebrate taxa were exposed to *L. sphaericus*. Results indicated few impacts on taxa categorized into functional groups.

### Secondary effects: larvicides and gene-drive mosquitoes

Although all substances are toxic depending on the dose, it is clear that proteins expressed in a gene-drive system to suppress or modify mosquito populations for malaria management would not be similar to larvicidal active ingredients. They would most likely be practically non-toxic to non-target organisms and would challenge the current situation with pesticides that there are deleterious effects other than those caused by a reduction in the population of the target population. Further, as proteins expressed in mosquito larvae, they would almost certainly be expressed at environmental concentrations that are orders of magnitude lower than conventional larvicides (Connolly et al. 2021).

Consequently, the focus in most cases would be on the secondary effects associated with population suppression of the target organism (in this case, species in the *An. gambiae* or *An. funestus* complex). It is important to note that the goal of both conventional larvicides and the gene-drive systems discussed here is to lower the population of the pest mosquito to reduce malaria (Fig. 1). Indeed, that is the point of the management tactic unless the focus is population modification. In the following paragraphs, we discuss secondary effects that apply to both current and gene-drive approaches.

### Immature mosquitoes as food for predators

One secondary effect of population suppression is the potential reduction of beneficial species that feed on the larvae and pupae of *An. gambiae* (sensu lato) (Fig. 1). Many invertebrate species and larvivorous fish feed on the aquatic larval and pupal life stages of mosquitoes (Service 1977; Ohba et al. 2010; Dida et al. 2015). Predatory invertebrates may be responsible for as much as 90% of the mortality of immature mosquitoes in certain aquatic habitats (Service 1971, 1973, 1977). In the wetlands of western Kenya, Ohba et al. (2010) found that 54.2% of 330 potential predators had ingested immature stages of *An. gambiae*, including Odonata larvae (70.2%), Hemiptera (62.8%), Amphibia (41.7%), and Coleoptera (18%).

However, there is little evidence that aquatic predators rely solely on immature mosquitoes for survival. Rather, larval and pupal stages of mosquitoes serve as one of many food sources for predators. After an extensive literature review of *An. gambiae* predation in Africa, Collins et al. (2019) suggested that no predators have been found to be closely associated or dependent on *An. gambiae* larvae, and that this mosquito complex is probably not an essential part of any ecosystem food web. Roberts et al. (2017) concurred, suggesting the loss of *An. gambiae* from a particular aquatic habitat is unlikely to cause ecological harm, even though many invertebrates and fish prey on this species. Likewise, Derua et al. (2018) found that long-lasting microbial larvicides (Bti and *L. sphaericus*), which reduce immature populations of *An. gambiae* and *An. funestus,* have no ecologically significant impact on the abundance or diversity of non-target invertebrates or vertebrates in the western highlands of Kenya.

Another important consideration for ecological risk is that in sub-Saharan Africa two of the three primary malaria vectors prefer small, ephemeral, sunlit water bodies that do not support predator populations (Carlson et al. 2004; Diabate et al. 2005; Gimonneau et al. 2010, 2012). Aquatic predators typically require more time to develop than mosquito larvae, and therefore occur in more permanent habitats (Kindlmann and Dixon 1999; Terhorst et al. 2010). Therefore, mosquito larvae in ephemeral habitats such as hoof prints or road ruts exhibit higher survival because there are fewer predators (Munga et al. 2006). The seasonality of *An. gambiae* combined with the ephemeral nature of its larval habitats likely results in predation that is limited to opportunistic generalist predators (Collins et al. 2019), and does not disproportionately and adversely affect any specific non-target species. Overall, the current weight of evidence suggests that a reduction in *An. gambiae* and closely related mosquito larvae most likely would have a negligible impact on predator abundance. Moreover, the species complex does not seem to play a key role in ecosystems (Collins et al. 2019; Connolly et al. 2021).

### Effects on the food of larval mosquitoes

Another secondary effect of population suppression could be an increase in algal blooms (including toxic algal blooms), which might adversely affect wildlife. Algae and other primary producers are important larval food sources for anopheline mosquitoes (Connolly et al. 2021). Kaufman et al. (2006) suggested that algal biomass on water surfaces is important for larval development of *An. gambiae*, and Gimnig et al. (2002) found that *An. gambiae* larval grazing reduced algal biomass and abundance in an experiment using an artificial habitat with rainwater seeded with cow dung. The presence of algal mats also serves as an attractant for ovipositing *Anopheles* females (Bond et al. 2005). Both *An. gambiae* and *An. funestus* have been positively associated with algae (Minakawa et al. 1999; Gimnig et al. 2001), despite their different habitat preferences. However, this association may also reflect the growth of inedible algal forms, such as filamentous green algae, which is indigestible for most invertebrates (Martin and Kukor 1984). Studies linking reductions in *An. gambiae* larvae to increases in algal blooms might be irrelevant because habitat used by this species is temporary and may not support healthy communities of primary producers (Teem et al. 2019). However, larvae of *An. coluzzii* and *An. funestus* occur in more complex, permanent habitats (Gimnig et al. 2001; Gimonneau et al. 2012) and might play a greater role in reducing algal blooms. Regardless, a decline in mosquito larvae would not affect toxic algal blooms because the cyanobacteria that comprise these blooms are toxic to many animals, including mosquito larvae, so cyanobacteria would not be reduced through feeding (Marten 2007; Connolly et al. 2021).

Bacteria, protozoa, and other primary producers may serve as secondary food sources for mosquito larvae and therefore may be affected by reduced numbers of larvae. Gimnig et al. (2002) suggested that if algal resources are depleted, *An. gambiae* larvae will feed on available bacteria, but bacterial abundance was not significantly affected. Östman et al. (2008) found that protozoan densities and diversity increased dramatically after floodwater mosquito populations were significantly reduced by Bti treatments.

Somewhat related to the food and detritus issue is the secondary effect of numerous dead *An. gambiae* larvae having a deleterious effect on water quality. To our knowledge, there have been no studies of this for current larvicides. Gene-drive population suppression would reduce the population, resulting in increasingly fewer larvae and therefore negate specific concerns about water quality due to extensive larval mortality. Conversely, with a larvicide, there would be dead larvae in the water and concentrations of the larvicide each time it is used.

### Effects of engineered proteins and nucleic acids

Another potential secondary effect is that dead gene-drive mosquito larvae will differentially contaminate the water compared to non-gene-drive larvae. Based on the techniques currently being investigated, it is unlikely that the DNA, RNA, or proteins responsible for population suppression in gene-drive mosquitoes would negatively affect water quality any more than non-gene-drive mosquitoes. Of course, the engineered proteins responsible for the desired effect in the gene-drive mosquitoes would be assessed for fundamental toxicity and allergenicity as is currently done with transgenic products, with positive toxicity or allergenicity likely leading to a regulatory rejection (EFSA 2010; EFSA et al. 2020; Connolly et al. 2021). Given the likely impact of the population suppression strategies, which would be to reduce the production of offspring (i.e., larvae), the “contamination” due to gene-drive larvae would be less than that of non-gene drive larvae, or gene-drive larvae from population modification strategies. However, in none of the larval types would the effect of the “contamination” be any greater than that of non-genetically engineered mosquitoes in the environment.

### Niche replacement

A substantive reduction of larval *An. gambiae* populations could also result in an ecological niche opening up for other vector species that transmit malaria or other diseases. Studies have documented mosquito management which reduced populations of anopheline mosquitoes in East Africa and resulted in higher densities of other species, likely because of preferential elimination of adults and consequently population reduction (Gillies and Smith 1960; Gillies and Furlong 1964; Bayoh et al. 2010). *Anopheles gambiae* is the most efficient vector of malaria (Lindsay et al. 1998), in part because it has a very effective biological response to competition. It reduces its larval developmental time in the presence of competitors without an increase in larval mortality or a reduction in body size, but the effect depends on water volume (Paaijmans et al. 2009). This strategy results in higher competitive success compared to *An. arabiensis* or *An. coluzzii*, which share aquatic habitats with *An. gambiae* but have lower rates of malaria transmission. Therefore, any reduction in *An. gambiae* abundance should translate to reduced risk of malaria, since the competitors most likely to replace it are not as efficient vectors.

The *An. gambiae* complex is comprised of many morphologically indistinguishable species, which means hybridization potentially occurs. If gene flow between species includes the gene construct of gene-drive mosquitoes, malaria transmission may be further reduced, as naïve species in the complex are exposed and eventually genetically modified (Roberts et al. 2017). Under such conditions, this management tactic should result in fewer inputs over time, including potentially requiring fewer larvicide applications. In addition, McArthur et al. (2014) determined that gene-drive *An. gambiae* larvae have the same mortality rate as wild-type larvae, suggesting there should not be an increase in the accumulation of phenotypes in the environment.

## Conclusion

Because of workshops with stakeholders that identified concerns about aquatic environments and water quality, we have used a comparative qualitative risk assessment approach for aquatic environments (Roberts et al. 2017; Teem et al. 2019; Connolly et al. 2021). We conclude that the tactic of gene-drive *An. gambiae* for malaria management is unlikely to result in risks to aquatic environments that exceed current larviciding tactics. Although these systems currently are in research and development stages, it is likely that the resulting proteins will not have insecticidal properties that are mechanistically similar to current larvicides. As such, they should not impose risks on water quality and non-target aquatic organisms that are greater than current larvicides. In fact, the risks might be lower (Fig. 2). Our conclusions directly relate to the important regulatory concept of “substantial equivalence” (Codex Alimentarius Commission 2003). Furthermore, they are consistent with the recommended safety standard of James et al. (2020), who recommend that gene-drive mosquitoes should be released only if they “…will do no more harm to human health than wild-type mosquitoes of the same genetic background and no more harm to the ecosystem than other conventional vector control interventions.”

It is important to reiterate, however, that in most cases gene-drive mosquitoes will be used within an existing IPM system. Consequently, IPM tactics such as larvicides and gene-drive mosquitoes will be used concurrently and regulators will need to ensure that risks from all tactics are acceptable.

Traditionally, risk assessment relies on estimating or using actual environmental exposures to the stressor and comparing those to effects to arrive at a quantitative characterization of risk. However, for gene-drive mosquitoes, the problem formulation, stressor identification, and effect assessment may be more important to the final risk estimate (Fig. 2), especially in these early days when there is no body of experiential use data.

Mosquito and malaria management should always use IPM. This approach is also referred to as Integrated Mosquito Management (IMM) and Integrated Vector Management (IVM) when concerned with mosquito vector management. IPM is a comprehensive approach to managing pests that is economically and ecologically sustainable (Peterson et al. 2018). Although using multiple tactics and integrating those tactics are not an absolute requirement for a successful, sustainable IPM program, they are commonly a feature of IPM. The concept of ecological sustainability includes resistance by the pest to the management tactic, and, therefore, an overall goal of IPM is to manage resistance. This is especially salient because management tactics such as contemporary synthetic insecticides, biological insecticides, and gene-drive approaches obviate long-term issues of broad-spectrum toxicity and environmental residuality of pesticides. Consequently, because resistance development by pests is arguably the most significant contemporary risk with management tactics (Peterson et al. 2018), the discovery and development of new tactics is critical to long-term management success (WHO 2020). Provided initial regulatory safety assessments and field applications and monitoring are successful, gene-drive mosquitoes will undoubtedly be an important tactic within IPM programs for malaria management.
