# Correction: Larval mosquito management and risk to aquatic ecosystems: A comparative approach including current tactics and gene-drive *Anopheles* techniques

**DOI:** 10.1007/s11248-022-00317-7

**Published:** 2022-08-09

**Authors:** Robert K. D. Peterson, Marni G. Rolston

**Affiliations:** Department of Land Resources & Environmental Sciences, Montana State University, Bozeman, MT 59717-3120 USA

## Correction to: Transgenic Res https://doi.org/10.1007/s11248-022-00315-9

The article “Larval mosquito management and risk to aquatic ecosystems: A comparative approach including current tactics and gene-drive *Anopheles* techniques”, written by Robert K. D. Peterson and Marni G. Rolston, was originally published electronically on the publisher’s internet portal on 7 July 2022 without open access. With the author(s)’ decision to opt for Open Choice the copyright of the article changed on 26 July 2022 to © The Author(s) 2022 and the article is forthwith distributed under a Creative Commons Attribution 4.0 International License, which permits use, sharing, adaptation, distribution and reproduction in any medium or format, as long as you give appropriate credit to the original author(s) and the source, provide a link to the Creative Commons licence, and indicate if changes were made.

The images or other third party material in this article are included in the article’s Creative Commons licence, unless indicated otherwise in a credit line to the material. If material is not included in the article’s Creative Commons licence and your intended use is not permitted by statutory regulation or exceeds the permitted use, you will need to obtain permission directly from the copyright holder.

To view a copy of this licence, visit http://creativecommons.org/licenses/by/4.0/.

